# Imaging in idiopathic pulmonary fibrosis: diagnosis and mimics

**DOI:** 10.6061/clinics/2019/e225

**Published:** 2019-01-25

**Authors:** Bruno Hochhegger, Edson Marchiori, Matheus Zanon, Adalberto Sperb Rubin, Renata Fragomeni, Stephan Altmayer, Carlos Roberto Ribeiro Carvalho, Bruno Guedes Baldi

**Affiliations:** I Departamento de Radiologia, Laboratorio de Pesquisas em Imagens Medicas (LABIMED), Irmandade Santa Casa de Misericordia de Porto Alegre, Universidade Federal de Ciencias da Saude de Porto Alegre, Porto Alegre, RS, BR; II Departamento de Radiologia, Universidade Federal do Rio de Janeiro, Rio de Janeiro, RJ, BR; III Departamento de Pneumologia, Irmandade Santa Casa de Misericordia de Porto Alegre, Universidade Federal de Ciencias da Saude de Porto Alegre, Porto Alegre, RS, BR; IV Departamento de Patologia, Universidade Federal de Ciencias da Saude de Porto Alegre, Porto Alegre, RS, BR; V Divisao Pulmonar, Instituto do Coracao (InCor), Hospital das Clinicas HCFMUSP, Faculdade de Medicina, Universidade de Sao Paulo, Sao Paulo, SP, BR

**Keywords:** Differential Diagnosis, Idiopathic Pulmonary Fibrosis, Radiology, Pathology, Interstitial Lung Diseases

## Abstract

Idiopathic pulmonary fibrosis is a chronic disease of unknown etiology that usually has a progressive course and is commonly associated with a poor prognosis. The main symptoms of idiopathic pulmonary fibrosis, including progressive dyspnea and dry cough, are often nonspecific. Chest high-resolution computed tomography is the primary modality used in the initial assessment of patients with suspected idiopathic pulmonary fibrosis and may have considerable influence on subsequent management decisions. The main role of computed tomography is to distinguish chronic fibrosing lung diseases with a usual interstitial pneumonia pattern from those presenting with a non-usual interstitial pneumonia pattern, suggesting an alternative diagnosis when possible. A usual interstitial pneumonia pattern on chest tomography is characterized by the presence subpleural and basal predominance, reticular abnormality honeycombing with or without traction bronchiectasis, and the absence of features suggestive of an alternative diagnosis. Idiopathic pulmonary fibrosis can be diagnosed according to clinical and radiological criteria in approximately 66.6% of cases. Confirmation of an idiopathic pulmonary fibrosis diagnosis is challenging, requiring the exclusion of pulmonary fibroses with known causes, such as asbestosis, connective tissue diseases, drug exposure, chronic hypersensitivity pneumonitis, and other forms of idiopathic interstitial pneumonitis. The histopathological hallmark of usual interstitial pneumonia is a heterogeneous appearance, characterized by areas of fibrosis with scarring and honeycombing alternating with areas of less affected or normal parenchyma. The aim of this article was to review the clinical, radiological, and pathological features of idiopathic pulmonary fibrosis and of diseases that might mimic idiopathic pulmonary fibrosis presentation.

## INTRODUCTION AND HISTORY

Idiopathic pulmonary fibrosis (IPF) is a chronic disease of unknown etiology that usually has a progressive course, and IPF is commonly associated with a poor prognosis. Although IPF has been described extensively in the literature, some controversy exists regarding its history. The first description of IPF as a new clinical and pathological entity is usually attributed to a 1933 report by Hamman & Rich 1. Nevertheless, many reports in German have previously published descriptions of autopsy findings that were consistent with IPF 2. Patients presented with progressively worsening cough and dyspnea, and autopsies usually revealed hypertrophied right ventricles, small rigid lungs with widened and thickened bronchioli, and an increased quantity of interstitial tissue without pleural adhesions 2. In these German reports, different terms were proposed to describe these interstitial changes, namely, *cirrhosis cystica pulmonum* and *lymphangitis reticularis pulmonum* 2.

### Clinical and laboratory assessment

A detailed clinical assessment is essential for the diagnosis of patients with interstitial lung diseases (ILDs) and for the diagnostic confirmation of IPF. A detailed investigation of exposure to external agents, such as mold, birds, and drugs, should be performed. Evidence of extrapulmonary manifestations, such as arthralgia, Raynaud phenomenon, dry mouth and eyes, and skin lesions, are essential to the approach for ILDs as these factors can be helpful in establishing the diagnosis of connective tissue diseases (CTDs), which can also present a usual interstitial pneumonia (UIP) pattern. An investigation of the family history of lung disorders is also recommended because CTDs and hereditary diseases are potential etiologies of ILDs 3-5.

IPF mainly affects patients in their sixth and seventh decades of life, with a higher prevalence in males and smokers or former smokers, and IPF affects the lungs exclusively 3. Gastroesophageal reflux is a common association 3,6.

The main symptoms of IPF, including progressive dyspnea and dry cough, are often nonspecific 6. Frequent signs on physical examination include the presence of bilateral inspiratory crackles (Velcro-like) predominantly in the lower lung zones, and digital clubbing 3,5. Pulmonary function tests (PFTs) in IPF are characterized by a restrictive pattern combined with a decreased diffusing capacity. Diminished exercise performance and hypoxemia at rest or during exercise may be found 7.

Serological analyses, including tests for rheumatoid factor (RF), anti-cyclic citrullinated peptide, and anti-nuclear antibody (ANA), are helpful in the differential diagnosis as the UIP pattern can also be found in CTDs 7,8. However, mildly positive ANA and/or RF serology can be found in IPF 5.

### Computed tomography signs (definition, accuracy, interobserver agreement and differential diagnosis)

A ground-glass opacity (GGO), a reticular pattern, traction bronchiectasis, and honeycombing are among the most common features of ILDs on high-resolution computed tomography (HRCT), and physicians should be familiar with the definitions, accuracies, and differential diagnoses of these features for the diagnostic work-up.

### Ground-glass opacity

**Figure 1:**
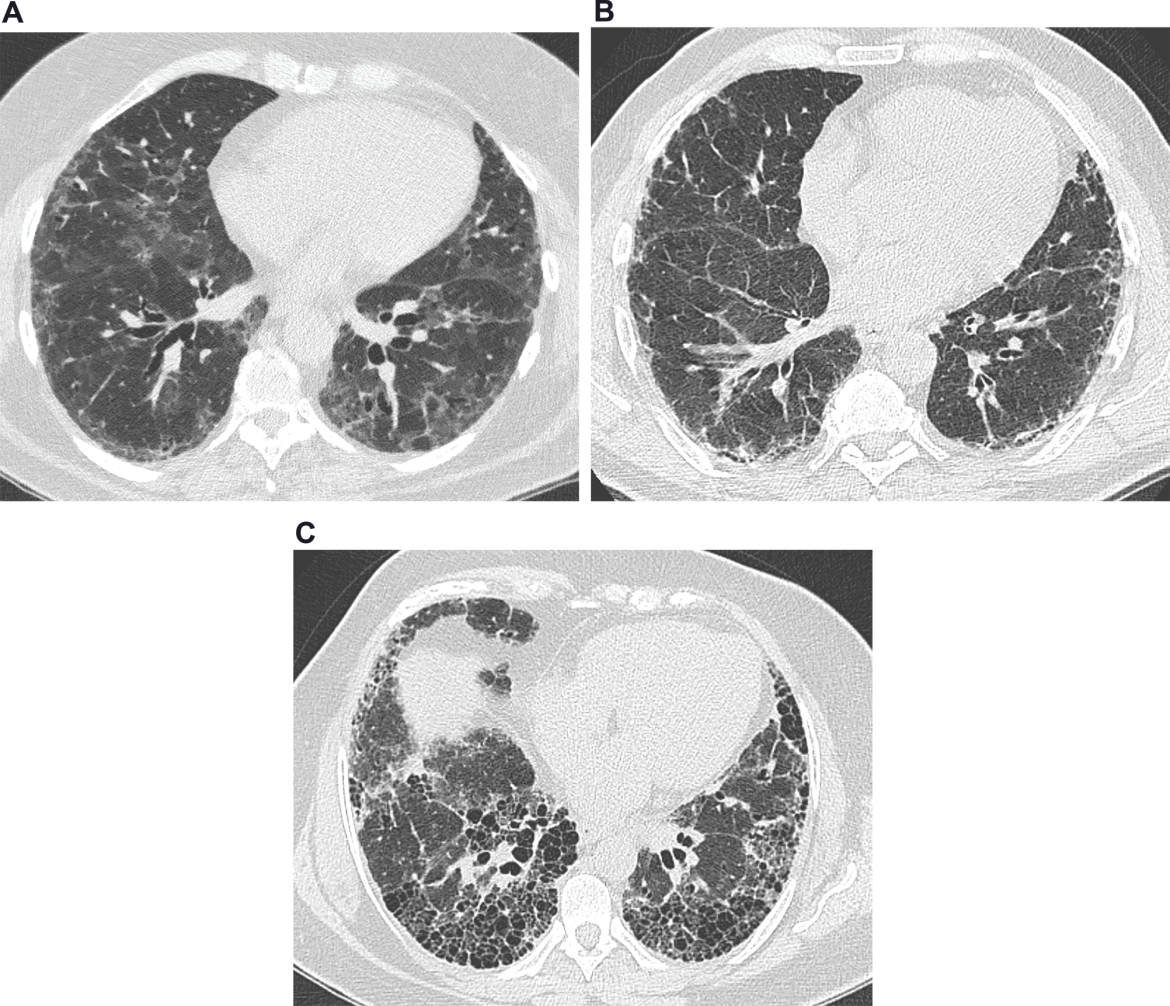
Common features on high-resolution computed tomography in interstitial lung diseases. **(a)** Images from a 63-year-old female presenting a nonspecific interstitial pneumonia pattern. There are predominant areas of ground-glass opacities, with some traction bronchiectasis and cortical interlobular septal thickening. **(b)** Images from a 61-year-old male with idiopathic pulmonary fibrosis. There are diffuse areas of interlobular septal thickening, predominantly in the cortical lung zones. **(c)** Images from a 56-year-old female with idiopathic pulmonary fibrosis. There are extensive areas of honeycombing, with some interlobular septal thickening.

On computed tomography (CT) imaging, GGO presents as a dense area of increased opacity within the lungs that conserves bronchial and vascular margins (Figure 1A) 9. GGO is less hazy than consolidation, in which bronchovascular margins cannot be distinguished. GGO can be due to the partial filling of airspaces, interstitial thickening (as a result of fluid, cells, and/or fibrosis), the partial collapse of alveoli, an increased capillary blood volume, or a combination of these, whereas all are related to the common partial displacement of air 9. Good interobserver agreement has been reported in the detection of GGO (kappa value, 0.78-0.90) 10.

### Reticular pattern

A reticular pattern is defined as a collection of several small linear opacities that resemble a net-like aspect (Figure 1B) 9. The components of a reticular pattern are clearly observed on thin-section CT, and they can represent interlobular septal thickening, intralobular lines, or the cyst walls of honeycombing. This finding is usually associated with ILD, but congestion and infections (e.g., viral) are also important differential diagnoses 9.

### Honeycombing

Honeycombing is defined as clustered cystic airspaces that are usually subpleural with well-defined walls and diameters ranging from 0.3-1 cm, reaching 2.5 cm is rare cases (Figure 1C) 9. Commonly considered specific for IPF, honeycombing is an essential criterion for UIP diagnosis, and the terminology should be used carefully as it may directly influence patient management 11. Centrilobular emphysema, traction bronchiectasis, and cystic lung disease should be included in the differential diagnoses. Interobserver agreement for honeycombing is moderate (kappa=0.59±0.12). In a study by Watadani et al., there was disagreement on the identification of honeycombing in 29% of cases due to the co-existence of traction bronchiectasis, large cysts, and overlapping pulmonary emphysema 12.

### Traction bronchiectasis

Traction bronchiectasis and bronchiolectasis represent nonuniform bronchial and bronchiolar dilatation, respectively (Figure 1A) 9. Dilated airways can also present as cysts (bronchi) or microcysts (bronchioles in the lung periphery). In IPF, traction bronchiectasis is better explained as a result of bronchiolar proliferation instead of utter mechanical traction 13. Recent studies have suggested that traction bronchiectasis and honeycombing are parts of a spectrum of the presentation of a singular and continuous mechanism of bronchiolar dysplastic proliferation in IPF 13,14. On the other hand, in nonspecific interstitial pneumonia (NSIP), bronchocentricity is predominant, and traction bronchiectasis is exclusively surrounded by fibrotic tissue, characteristics that suggest the mechanical traction as the main component in the development of traction bronchiectasis in NSIP 13,14. Interobserver agreement for traction bronchiectasis is moderate, with kappa舁values ranging from 0.24 to 0.42 11.

### Idiopathic pulmonary fibrosis

#### Computed tomography diagnostic criteria (usual interstitial pneumonia)

Chest HRCT plays an essential role in the initial assessment of suspected IPF, considerably influencing subsequent management decisions. The primary role of HRCT is to distinguish chronic fibrosing lung diseases with an UIP pattern from those presenting a non-UIP pattern, suggesting an alternative diagnosis when possible. The most common HRCT protocol used to evaluate diffuse lung diseases is a volumetric acquisition of thin sections (generally <1.5 mm), combined with a high spatial frequency reconstruction algorithm 15. The 2018 ATS/ERS/JRS/ALAT guidelines for diagnosis of IPF 6 suggested four diagnostic entities: (a) UIP, (b) probable UIP, (c) indeterminate for UIP, and (d) alternative diagnosis (Table 1). The HRCT criteria for a UIP diagnosis include the presence of honeycombing with or without peripheral traction bronchiectasis or bronchiolectasis and a basal and subpleural distribution 6. Although GGO is common in UIP, it must be less extensive than the reticulation and be superimposed on a fine reticular pattern to be characterized as an UIP pattern 6. Another common feature on HRCT is mediastinal and hilar lymph node enlargement, which is present in up to 70% to 86% of patients with a UIP pattern (typically <15 mm) 16.

In approximately 66.6% of cases, a diagnosis of IPF can be performed based only on clinical and radiological features 17. The morbidity and mortality associated with lung biopsies in patients with fibrosis are high, at approximately 3-4% in most studies 18. Several recent reports suggest that surgical lung biopsy is unnecessary for an IPF diagnosis in some cases with possible UIP patterns on HRCT in the appropriate clinical settings at specialized centers 19. A confident diagnosis of UIP based on HRCT is accurate in 80% to 95% of cases using pathology as a reference 20. In multiple studies assessing the CT accuracy for an IPF diagnosis, pathology was considered a gold standard. However, there is substantial interobserver variation among pathologists in the assessment of non-neoplastic lung disease 21. An interobserver variation over 50% has been reported for the pathological diagnosis of NSIP and its distinction from UIP 21.

#### Histopathology diagnostic criteria (usual interstitial pneumonia)

**Figure 2:**
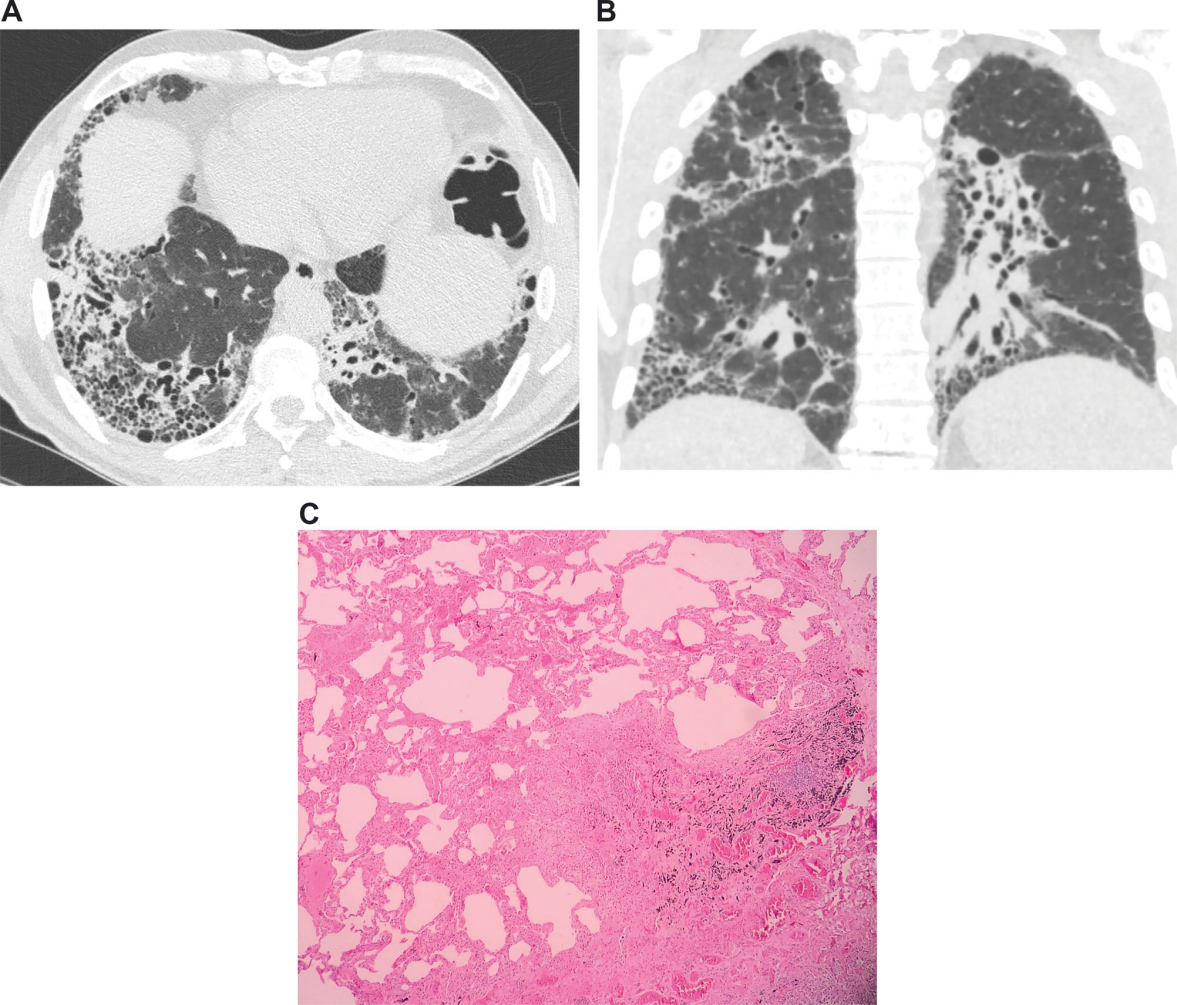
Images from a 53-year-old male with idiopathic pulmonary fibrosis. **(a)** Axial and **(b)** coronal computed tomography images demonstrating areas of honeycombing, reticulation and subpleural predominance. **(c)** Histopathology images demonstrating areas of marked fibrosis, with architectural distortion and fibroblast foci, alternating with areas of normal parenchyma.

The main histopathologic feature and diagnostic criterion of UIP is a patchy presentation comprising affected zones of fibrosis with scarring and honeycombing alternating with zones of less affected or healthy tissue (Table 2) (Figure 2C) 22. These findings usually prevail in the subpleural and paraseptal parenchyma. Inflammation is commonly absent or mild, consisting of an irregular interstitial infiltrate of lymphocytes and plasma cells associated with the hyperplasia of type 2 pneumocytes and the bronchiolar epithelium. Although the fibrotic areas consist primarily of dense collagen, the diffuse convex subepithelial foci of proliferating fibroblasts and myofibroblasts are typical findings (fibroblast foci). These should be differentiated from fibroblastic alveolar plugs of organizing pneumonia. While the former is always within the interstitial spaces, the latter is associated with polypoid intrusions into the alveolar space. Zones of honeycombing are formed by cystic fibrotic airspaces that are commonly lined by a bronchiolar epithelium and filled with mucus and inflammatory cells 22.

#### Pathogenesis

The pathogenesis of IPF has been discussed for several years, and one of the most accepted views is that the initial inflammatory events might initiate a dysregulated fibroblast-mediated wound-healing response that can be sustained by several factors, leading to lung fibrosis 23. One of the most accepted models is the “alveolar stem cell exhaustion” model, which suggests that an accelerated parenchymal senescence that is determined by predisposing factors, such as genetic mutations or variations and telomerase dysfunction, together with environmental exposures, such as tobacco smoke, can severely compromise the regenerative potential of parenchymal epithelial stem cells 13,24,25. This epithelial damage to the airway wall can induce alveolar collapse, what can trigger a fibroproliferation process and promote the destruction of airways, eventually leading to severe and irreversible functional impairment 24-26.

Mai et al. 2016 recently used micro-computed tomography to analyze the geometric progression of lung changes caused by IPF, associating the analysis concomitantly with histopathological and CT findings, and the authors encountered evidence suggesting that the disease started preferentially at the peripheries of the secondary pulmonary lobules, gradually extended toward the centrilobular region, and ultimately replaced the entire lobule 26. The sequence of lung changes on CT in patients with IPF starts with areas of GGO that turn into areas of reticulation and end with cystic changes 26,27. The so-called “microscopic honeycombing” represents the formation of these cystic spaces. These spaces should be differentiated from the honeycombing visualized in CT scans; these spaces are present in most cases and are not necessarily specific to a UIP diagnosis, while honeycombing is highly specific to UIP and is associated with a poor prognosis 26,28.

#### When is biopsy necessary?

Surgical lung biopsy (SLB) is the diagnostic method of choice for patients whose imaging findings are not definitive for UIP, including findings of probable UIP, indeterminate for UIP and alternative diagnosis. Hence, an integrated multidisciplinary approach assessing both imaging and pathological features is essential to establish a final diagnosis 3,6.

Tissue samples should be obtained from two to three lobes due to the high variability in the distribution and morphology of abnormalities. Careful evaluation of the presence of potential risks before performing SLB is essential as the risks associated with the procedure can outweigh the benefits of determining a diagnosis, especially in older patients, in those with severe impairment in PFTs or those with co-existing comorbidities, such as pulmonary hypertension and severe heart failure 6,29. Recent evidence suggests that cryobiopsy is a promising method that is less invasive than SLB and can be used in place of SLB to obtain lung samples to confirm an IPF diagnosis 30.

#### Mimics of idiopathic pulmonary fibrosis

##### Clinical mimics

Confirmation of an IPF diagnosis is challenging, requiring the exclusion of pulmonary fibrosis with known causes, such as asbestosis, CTDs, drug exposure, chronic hypersensitivity pneumonitis (HP), and other forms of idiopathic interstitial pneumonitis 3,5,6. Although these entities have similarities with IPF, such as progressive dyspnea, dry cough, and a restrictive pattern with a decreased diffusing capacity in the PFT, they are associated with better prognosis and an improved responsiveness to immunosuppressive drugs 7.

###### Connective tissue diseases.

These disorders often affect young women, and rheumatoid arthritis and systemic sclerosis are the most common CTDs associated with the UIP pattern. Extrapulmonary manifestations, such as arthralgia, Sicca symptoms, Raynaud phenomenon, and esophageal manifestations, are commonly identified in ILD associated with CTD. However, interstitial lung changes can be the sole manifestation of CTD. The presence of serological abnormalities is an important feature of CTDs 7. ILD is also associated with interstitial pneumonia with autoimmune features, which should be included in the differential diagnosis of IPF 31,32.

###### Hypersensitivity pneumonitis.

HP is an inflammatory syndrome associated with an exposure history to suspected antigens, such as mold, birds and drugs. Hence, potential exposures should be extensively investigated in patients with pulmonary fibrosis. Fatigue, fever, and weight loss may be found in acute and subacute HP and rarely in those with chronic forms. Progressive dyspnea is the main symptom in chronic HP, and inspiratory squeaks on pulmonary auscultation and digital clubbing may be identified. If the responsible inhaled antigen can be identified, complete avoidance is the most efficient management 5,29. Acute disease is commonly self-limited, resolving without any specific therapy. Corticosteroid therapy might be necessary for an acute relief of symptoms and can hasten initial recovery in severe cases. However, long-term efficacy has not been demonstrated in prospective clinical trials 33.

###### Idiopathic nonspecific interstitial pneumonia.

Compared with IPF, idiopathic NSIP occurs predominantly in young women and can have associated systemic manifestations, such as fever, fatigue, and weight loss, and features suggestive of CTD 4,7,34.

###### Asbestosis.

The main feature for distinguishing asbestosis from IPF is a history of occupational exposure to asbestos, such as asbestos mining, shipbuilding, and welding. Determining a temporal association between the exposure and the occurrence of symptoms is essential 7.

###### Drug-induced lung diseases.

Investigations of exposures to drugs that can induce ILD, such as bleomycin, amiodarone, nitrofurantoin, methotrexate, and cyclophosphamide, is essential in the assessment of patients with a suspicion of IPF. Determining a temporal relationship between the exposure to the potential drug and the occurrence of respiratory manifestations is also necessary 5.

##### Radiological mimics

NSIP accounts for most of the disagreements among observers for idiopathic interstitial pneumonias. HRCT findings that favor NSIP rather than UIP include the presence of GGOs, which are found in most cases. Subpleural sparing is highly suggestive of NSIP and is present in 64% of cases compared to 11% in chronic HP and 4% in IPF 34. The features that best differentiate chronic HP from IPF and NSIP are lobular zones with decreased attenuation, the presence of centrilobular nodules, and a lack of a lower zone predominance of abnormalities. The former has been reported in 80% of patients with chronic HP, 43% of patients with IPF, and 34% of patients with NSIP 34. The UIP pattern on HRCT in patients with CTDs or fibrotic HP is similar to the pattern found in idiopathic UIP, and this should be distinguished on a clinical basis. However, radiologists may contribute to such differentiation based on additional findings, such as esophageal dilatation, and on pleural or pericardial effusion or thickening. Asbestosis typically has a histologic and HRCT pattern of UIP, and pleural plaques usually aid in the diagnosis. Fibrotic sarcoidosis can be distinguished from UIP by the upper lobe, a predominant peribronchial distribution and other HRCT findings, including pulmonary nodules, lymph node enlargement and calcifications.

##### Histopathological mimics

During an examination of an ILD patient biopsy, the goal of the pathologist is to differentiate UIP from other diseases that may simulate this pattern, such as CTDs, chronic HP, pneumoconiosis, and NSIP 7,35.

###### Connective tissue diseases.

CTDs are a heterogeneous group of diseases, with different patterns on biopsy. Although the most common pattern is an NSIP pattern, UIP is frequently observed. The presence of an exuberant lymphocytic infiltrate that forms germinal centers and of follicular bronchiolitis can be helpful features for CTD diagnosis in these situations. In addition, the presence of pleural fibrosis is more characteristic of CTDs 7,35.

###### Chronic hypersensitivity pneumonitis.

In chronic HP, fibrosis is predominantly airway centered but can occasionally manifest as UIP. Features that might suggest a diagnosis of chronic HP include a predominance of upper lobes, the presence of interstitial granulomas, which are mainly peribronchiolar, and bronchiolitis with peribronchiolar metaplasia 7,35. Recent studies focusing on genetic factors associated with chronic HP have suggested a common pathobiology with IPF. A MUC5B rs35705950 single-nucleotide polymorphism and a short telomere length, as seen in sporadic and familial forms of IPF, can predispose patients with HP to lung remodeling and fibrosis 36.

###### Asbestosis.

Asbestosis is histopathologically characterized by the presence of predominantly subpleural interstitial fibrosis, which is similar to UIP. However, in early cases, fibrosis can be more prominent around bronchioles. Other helpful features in the differential diagnosis with UIP are collagen fibrosis with the absence of fibroblastic foci and a mild inflammatory infiltrate. To confirm the histological diagnosis, asbestos bodies must be present 37,38.

###### Nonspecific interstitial pneumonia.

NSIP is a type of chronic interstitial pneumonia that is characterized by a relative spatial homogeneity in the involvement of lung parenchyma, which is different from the typical patchy pattern of UIP where regions of the normal lung are interspersed with the affected parenchyma. In addition, NSIP is characterized by a temporal homogeneity of lesions (inflammation and/or fibrosis), which is different from the typical temporal heterogeneity of UIP 7,35,. Other features of UIP, such as smooth muscle hyperplasia, fibroblastic foci, and an aggregation of elastic fibers, are not observed in most cases of NSIP 39,40. In addition, peripheral accentuation is absent or inconspicuous 41.

Although the UIP pattern may be observed in several ILDs, there are relevant clinical, tomographic, and histopathological features that contribute to differentiating them from IPF. The diagnosis of IPF is challenging, and a multidisciplinary discussion with ILD experts, including physicians, radiologists, and pathologists, improves the accuracy of the diagnosis, which is essential to establishing the appropriate management strategy.

## Figures and Tables

**Table 1:** HRCT Criteria for the UIP Pattern.

UIP	Probable UIP	Indeterminate for UIP	Alternative diagnosis
- Predominantly subpleural and basal distribution - Reticular abnormality - Honeycombing with or without traction bronchiectasis	- Predominantly subpleural and basal distribution - Reticular abnormality with peripheral traction bronchiectasis - May have mild GGO	- Predominantly subpleural and basal distribution - Mild reticulation, mild GGO or distortion - CT features that do not suggest any specific etiology	- Distribution other than subpleural/basal - Extensive GGO (extension > reticular abnormality) - Profuse micronodules (bilateral, predominantly upper lobes) - Discrete cysts (multiple, bilateral, apart from areas of honeycombing) - Diffuse mosaic attenuation/air-trapping (bilateral, in three or more lobes) - Consolidation in bronchopulmonary segment(s)/lobe(s)

CT: Computed Tomography; GGO: Ground-glass Opacity; UIP: Usual Interstitial Pneumonia; >: Greater Than.

Adapted from Raghu et al. (6).

**Table 2:** Histopathological Criteria for the UIP Pattern.

UIP	Probable UIP	Indeterminate for UIP	Alternative diagnosis
- Evidence of marked fibrosis / architectural distortion, ± honeycombing in a predominantly subpleural / paraseptal distribution - Patchy involvement of lung parenchyma by fibrosis - Fibroblast foci	- Some characteristics of column 1 but not sufficient to corroborate the diagnosis of UIP- Exclusively honeycombing changes - Absence of features to suggest an alternative diagnosis	- Some histologic features of column 1 associated with features that suggest another diagnosis or UIP secondary to another cause (granulomas, hyaline membranes, cellular inflammatory infiltrate in areas away from honeycombing, prominent lymphoid hyperplasia, bronchiolocentric distribution, exuberant chronic fibrous pleuritis and organizing pneumonia)	- Hyaline membranes - Organizing pneumonia - Granulomas - Marked interstitial inflammatory cell infiltrate away from honeycombing - Predominant airway-centered changes - Other features suggestive of an alternate diagnosis

UIP: Usual Interstitial Pneumonia.

Adapted from Raghu et al. (6).
